# The Development of a Pilot App Targeting Short-Term and Prospective Memory in People Diagnosed with Dementia

**DOI:** 10.3390/bs13090752

**Published:** 2023-09-11

**Authors:** Vicky Nanousi, Konstantina Kalogeraki, Aikaterini Smyrnaiou, Manila Tola, Foteini Bokari, Voula Chris Georgopoulos

**Affiliations:** 1 Department of Speech and Language Therapy, School of Health Rehabilitation Sciences, University of Patras, 26504 Patras, Greece; 2 Primary Health Care Laboratory, School of Health Rehabilitation Sciences, University of Patras, 26504 Patras, Greece

**Keywords:** dementia, short-term memory, prospective memory, rehabilitation of dementia, technological devices, apps

## Abstract

Background: According to the World Health Organization, people suffering from dementia exhibit a serious decline in various cognitive domains and especially in memory. Aims: This study aims to create a pilot computer app to enhance short-term memory and prospective memory in individuals with dementia using errorless learning based on their individualized needs. Methods: Fifteen dementia patients and matched controls, matched for age, sex, and education, were selected. Their daily routines were analyzed, and cognitive abilities were assessed using the MoCA test. Considering the participants’ illness severity and daily needs, the pilot app was designed to aid in remembering daily tasks (taking medication and meals), object locations, and familiar faces and names. Results: An improvement in patients’ short-term and prospective memory throughout the training sessions, but not in overall cognitive functioning was observed. A statistically significant difference between patients and healthy controls was indicated in their ability to retain information relevant to them in their short-term memory, or to remember to act in the future following schedules organized at present (*p* < 0.001). Conclusion: This app appears beneficial for training dementia patients and healthy individuals in addressing memory challenges. Recommendation: While the pilot app showed promise, further research with larger samples is recommended.

## 1. Introduction

Currently, over 55 million people worldwide are affected by dementia [1]. It is a disease that constitutes ‘one of the main causes of incapability and dependency of older people’ as it interferes with normal daily functioning [2]. Dementia is a syndrome caused by several diseases (e.g., tumors, metabolic disorders, neurodegenerative diseases, etc.) typically affecting people over the age of 65 [1]. Alzheimer’s disease (AD) is the most prevalent form of dementia, responsible for roughly 60% of cases, followed by cerebrovascular dementia, accounting for about 30% of cases [3]. Dementia with Lewy bodies and frontotemporal dementia (FTD) are less common [1]. As the aging population grows, dementia’s prevalence increases due to its strong association with age [4]. Dementia’s profound characteristic is a gradual loss of cognitive functioning. The defining characteristic of dementia is the gradual deterioration of cognitive function. Its most critical feature is the progressive decline in cognitive abilities, encompassing memory, thinking, attention, concentration, and the capacity to maintain daily living activities. It can also affect emotional control and social behavior [1].

Normal aging, however, also impacts global cognition, as indicated by numerous studies [5,6]. Though it is an older approach, it is still assumed that certain cognitive functions are affected during aging, particularly the speed of processing. This, in turn, affects memory, specifically working memory, which deteriorates as humans age [7]. In addition to memory, other cognitive functions are also compromised, such as language, executive functions, and perception [8]. Cognitive skills tend to change throughout adulthood, with certain mental activities peaking at different points in time [9].

The idea that aging does not uniformly impact all cognitive functions is rooted in a theory by Catell [10]. He differentiated between two primary categories of cognitive functions, classifying them as fluid and crystallized intelligence, both contributing to overall general intelligence. According to Catell, fluid intelligence is a general ability to discern and perceive relationships among various elements, whether they are novel or familiar. Fluid intelligence encompasses abilities that involve deliberate processing, including working memory, processing speed, abstract reasoning, and visuospatial reasoning [11,12]. These capacities are crucial for complex thinking, problem-solving, and everyday functioning and they do not depend on prior experiences [13]. Conversely, crystallized abilities draw upon previously acquired cognitive skills, general knowledge, and vocabulary [11,12].

Research suggests a positive correlation between fluid and crystallized intelligence in cognitively normal adults [13,14]. However, as individuals age, crystallized abilities appear to remain stable or even show improvement [15], unless cognitive impairment or dementia sets in [16]. In contrast, fluid cognitive functions exhibit a steep decline, typically commencing around the age of 50, affecting various domains such as memory, working fluency, attention, concentration, and more [16]. This decline in fluid intelligence is underpinned by neural changes, suggesting reduced responsiveness in a frontoparietal network, which may explain the decrease in cognitive abilities during normal aging [17]. Indeed, neuroimaging research indicates that specific neuroanatomical regions are affected by aging, resulting in a decline in overall cognitive functioning [18,19,20,21,22].

The disparity between fluid and crystallized intelligence serves as a predictive factor for abnormal cognitive decline. Fluid intelligence experiences significant deterioration compared to crystallized intelligence [14], and it also leads to the development of compensatory aging theories [23].

Regarding dementia, it is well-documented [24,25,26] that patients with dementia, especially those with AD, initially experience a decline in fluid functions, which serves as a predictive marker for disease progression itself [13]. Crystallized cognitive functions, on the other hand, remain relatively intact, reflecting the patients’ cognitive abilities before the onset of the disease [13]. Nonetheless, dementia entails a global decline in intellectual abilities, primarily affecting memory and at least one other cognitive function [27].

Considering the magnitude of cognitive decline and its impact on independent living, particularly in the context of increasing lifespans, it becomes imperative to identify effective treatments for enhancing cognitive function in people with dementia or those at risk of developing any form of dementia. Currently, two options are available for dementia treatment: pharmacological and non-pharmacological interventions (see [28,29] for a literature review). Pharmacological treatments typically involve cholinesterase inhibitors, the NMDA receptor antagonist memantine, antipsychotic drugs, among others [30]. Non-pharmacological treatments address the unmet needs of dementia patients stemming from the symptoms of dementia itself, including cognitive decline, communication difficulties, and the inability to live independently [30].

Pharmacological interventions have been employed to mitigate the symptoms of dementia and slow its progression. However, despite being the primary approach for managing dementia, these treatments do not offer a “cure” for the disease. The effectiveness of pharmacological interventions varies from person to person and does not yield long-term benefits for patients [31,32]. In the absence of a viable long-term pharmacological remedy and in light of the presence of cognitive, behavioral, and psychological symptoms, it becomes crucial to provide individuals with dementia, their families, and their caregivers with additional assistance and alternative strategies through non-pharmacological interventions [31,32]. The objective of such interventions is to enhance or sustain patients’ cognitive function, improve their daily quality of life, and address behavioral symptoms that often accompany memory decline [31]. Non-pharmacological treatments are considered more cost-effective and associated with fewer side effects than pharmacological options, making them a preferable emerging treatment avenue for dementia [31,33]. There are four types of non-pharmacological interventions suggested, with cognitive training being one of them [33].

Cognitive training is an intervention method that employs structured (usually repetitive) exercises aimed at improving or maintaining mental function [34]. It can be delivered either individually or in a group, focusing on specific cognitive skills [8]. Advances in technology have led to the emergence of computerized cognitive training (CCT). These cognitive training exercises are delivered through electronic devices, such as smart phones, tablets, and computers, and can be customized to each patient’s needs, mental status, and expectations since they do not require significant effort on the part of the patients and utilize familiar activities only [35]. Computer-based applications can provide instant monitoring and control for every user, as well as data collection and metrics for each action in the electronic environment, allowing for the estimation of a participant’s performance and overall progress [35]. Additionally, they offer accessibility to individuals facing mobility issues and difficulties in accessing healthcare resources, all at a relatively low economic cost [36]. Finally, the use of rich multimedia in electronic applications provides users with the opportunity to engage in enjoyable activities and may even motivate them to repeat the activity and transfer the acquired knowledge to real-life situations [35].

Considering that cognitive training, especially using electronic devices, is currently a part of non-pharmacological interventions targeting specific cognitive functions, particularly memory (specifically, fluid intelligence), this study aims to further investigate whether intervention with a computer-based program would improve the short-term and prospective memory abilities of patients suffering from dementia and their normal peers. The rationale for investigating these specific types of memory is to determine whether a failure to remember to execute planned actions either immediately or in the immediate future (prospective memory) can have an impact on patients’ daily routines and consequently affect their quality of life. While the investigation of prospective memory is not extensive, it has yielded valuable outcomes regarding its treatment in dementia [37,38,39,40]. To facilitate our patients’ performance, we employed an errorless strategy, proven effective for improving memory abilities [41,42,43]. This was achieved with an app that provides participants with a user-friendly environment to track their step-by-step performance. Furthermore, there have been no other studies in Greek that have examined the performance of both dementia patients and their matched normal controls through an electronic application on tasks that require the use of specific types of memory. Finally, we also investigated the performance of healthy older participants on memory tasks to further examine whether a possible memory problem (a problem with a particular fluid intelligence ability) can be improved, raising hopes for the development and implementation of technological devices for researching memory decline through cognitive intervention strategies (errorless learning) that have thus far been considered very effective.

## 2. Literature Review on Computerized Memory Assisting Technologies

### 2.1. Studies Employing Computer-Based Technology as a Means of Intervention in Healthy Older Adults

A number of studies investigated the positive effects of cognitive training in healthy older individuals [34,44,45,46,47]. For example, in the ACTIVE study (advanced cognitive training for independent and vital elderly) the main objective was to examine the effects of memory, reasoning, and speed-of-processing training. The participants were randomly assigned to groups and were compared to no-training controls. RCTs were conducted to compare training among different cognitive domains [44]. It was found that each targeted cognitive ability showed a significant improvement as compared to the baseline assessment, and this improvement lasted up to 2 years, as indicated by a post-intervention follow-up [44]. Sustained improvements in specific cognitive domains (memory and reasoning) that lasted up to 5 years after training were further highlighted [48]. Following this line of research additional evidence was provided for the benefits of cognitive training [45]. It was noted that cognitive training can improve cognitive functions of healthy older adults. The observed improvement lasted up to five years after the intervention had begun. The implications of these outcomes were that an improvement in cognitive functions has a positive impact on daily living skills and it can also alleviate depressive symptoms.

An overall improvement of global cognitive function has also been reported, especially after the application of CCT programs in healthy adults, targeting specific cognitive domains such as verbal memory, working memory, attention, etc. [36,49,50,51]. Recently in a pilot study [36] a computerized cognitive stimulation website named VIRTRAEL (“Virtual Training for the Elderly people”, n.d.) was developed. This is a free access platform that includes tests and exercises for the assessment of cognitive skills in older people, such as attention, learning, memory, and executive functions. It was noted that there was an overall improvement of these targeted cognitive functions and it was proposed that CCT is in effect a useful tool that can be used in ‘the fight against cognitive symptomatology associated with aging and neurodegenerative diseases’.

Despite these promising outcomes and the ongoing research carried out in this area, the positive role of CCT’s approaches warrants further consideration. The benefits of computerized cognitive training in healthy older adults are being addressed, but at the same time, the problem concerning the efficacy of this method needs to be further examined, since this issue ‘remains controversial in the scientific community, and both sides of the debate encourage further research’ [52]. With this in mind, the present study hopes to provide additional evidence regarding the possible cognitive gains after the application of a computerized training program in both healthy and demented adults.

### 2.2. Studies Employing Computer-Based Technology as a Means of Intervention and Rehabilitation of Patients with Dementia

So far, most studies using technological means to enhance cognitive function in patients with dementia have mainly focused on AD while other types of dementia have been considered to a lesser extent.

In earlier studies [53,54,55,56], the efficacy of computer-based technology in patients with dementia was investigated and the common assumption drawn from these studies was that there was an overall improvement in patients’ cognitive performance. In particular, an overall improvement in patients’ training performance was noted [53], along with an improvement regarding the immediate and delayed recall of objects and routes [54]. In addition, a significant improvement in patients’ cognitive functions such as verbal fluency and executive functions was observed [55]. Improvement in specific cognitive areas has similarly been reported [57] where there was a good performance in the speed of data processing in patients with dementia and an almost normal performance in the comprehension of verbal, short memory, and perception tasks compared to normal subjects’ performance. A study of 20 Korean-speaking patients found that the application of a systematic computer-based cognitive training program may have beneficial effects in various cognitive areas such as language, attention, calculation, verbal memory, and frontal function at least for a short period of time [58]. However, in a systematic review of 31 studies that applied cognitive training in patients with mild or moderate AD, it was suggested that despite the heterogeneity and the variability of the interventions used, the outcome of those studies is that cognitive training may lead to an improvement of global cognition of the patients, particularly when these training programs are longer in duration and more intensive in nature [59]. At the same time, it is suggested that shorter interventions that aim at specific cognitive areas may also lead to an improvement of those particular areas. Other studies further support that computer-based cognitive interventions have a positive effect in the cognitive function of dementia patients and may also target and improve specific cognitive areas [60,61,62].

In relation to the easiness that patients with dementia can use and employ computer-based technology in their daily routine, it was found that the application of a cognitive training protocol is superior to other therapies for the cognitive improvement of patients with dementia [56]. These intervention programs offer patients independence [63] and account for the involvement of patients in leisure activities that promote healthier behaviors [64]. Moreover, it has also been pointed out that it is important to tailor computer activities to each patient’s needs [65]. Similarly, the importance of the development of person-centered tablet programs was noted as an implementation of a new service in dementia care [66].

The utilization of information and communication technologies (ICTs) as tools to enhance the quality of life for both individuals without dementia and those affected by dementia has been investigated [67]. Furthermore, it has been emphasized that the use of technological devices plays a crucial role in promoting a more active lifestyle for people with dementia, benefiting both their physical and mental well-being. In the same vein, [68] concluded that since patients with dementia find their interaction with technology enjoyable, then only interesting and enjoyable activities should be employed along with innovative ways to deliver them to patients with dementia, ways that can mimic real-time communication.

Even though there are a significant number of studies highlighting the positive effects of technological interventions, there are still certain studies that seem to be quite skeptical about the overall benefits that these non-pharmacological methods may in effect have.

The efficacy of computer cognitive rehabilitation in patients with mild cognitive decline was tested and the results indicated that the overall computer-based cognitive training in patients with AD and mild cognitive decline is effective at least in delaying the continuous progression of cognitive impairment in AD [69].

With respect to Greek language and Greek-speaking subjects with dementia, a relevant study presents the results of a computer-based intervention program for people with AD for a period of one year [35]. These patients have been tested before and after each intervention program (pre-test and post-test). The authors compared these data in an effort to examine the way the program performs and at the same time to assess the cognitive skills that may be improved. It was suggested that the patients’ overall scores were preserved for this period of time and they also showed a slight improvement. The authors concluded that the application of the specific intervention program had positive effects on patients’ overall performance.

Overall, the outcomes of the preceding studies indicate that the application of technology is necessary for the development of programs that can improve not only the cognitive impairments of patients with dementia and slow down the progression of the disease, but also provide a better quality of life since it reinforces a level of autonomy that is really important for the patients both emotionally and interpersonally.

Further evidence for the validity of the intervention with cognitive training programs derives from a great body of neuroimaging studies that underlie the importance of the application of these programs in relation to the beneficial effects on the physiology of the brain which are beyond the scope of this work (see for example a review paper of task related fMRI studies [70]; also studies reviewing the importance of specific brain networks and the impact that computer-based training has on them [71,72,73,74]).

### 2.3. Studies Based on Errorless Learning

One of the most promising rehabilitation strategies that has gained much interest over the last two decades and that is often adopted within the field of dementia care is that of errorless learning. Errorless learning (EL) is an approach used in memory rehabilitation and has its roots in behaviorism and, specifically, in the principles of implicit learning and memory. This approach was initially used in the research of amnestic patients who suffered from impaired explicit memory but had an intact implicit memory [75]. EL relies on implicit memory in the sense that there is no need for a conscious retrieval of information as in explicit memory and the information to be acquired is obtained in a more passive way. Thus, EL minimizes the chance of error production during learning and maximizes the chance of encoding only correct information through modeling, immediate provision of the correct answer, etc. In the literature of EL, the bulk of studies support the effectiveness of this method as it is applied to a variety of clinical populations.

#### 2.3.1. Errorless Learning in Healthy Older Adults

Positive effects of errorless learning have been widely reported, mainly in the clinical population of amnesic patients. However, only two studies [75,76] have examined this technique in relation to cognitive decline in normal ageing. A comparison of the efficacy of errorless and errorful learning on memory performance in older people and young adults showed that there was an overall lower memory performance and flatter learning curves for older adults as compared to young adults, regardless of the task conditions [76]. However, the researchers found a superiority effect of errorless learning as compared to errorful learning, a finding that was equally evident in both groups. They suggested that the prevention of errors during learning results is essentially an effective strategy and may lead to better memory performance.

#### 2.3.2. Errorless Learning and Dementia

In a study involving participants with severe memory impairment [75], it was observed that they exhibited a higher capacity to learn items from word lists through errorless learning compared to an errorful control group. This advantage of EL was consistently observed in various case studies, encompassing learning object names, novel face-to-name associations, new facts, names of rehabilitation ward staff, orientation information items, and methods for programming memory aids [77]. These findings were further substantiated by subsequent research conducted with populations of individuals with dementia and those in the early stages of AD, yielding similar outcomes [41,78,79].

Although there may not always be unanimous agreement among researchers regarding the benefits of EL, it is noteworthy that a majority of studies emphasize the efficacy of EL procedures in ameliorating memory difficulties in individuals with dementia. For instance, a review encompassing 26 studies involving individuals with dementia, all employing EL rehabilitation strategies, revealed that even individuals with minimal to moderate dementia can successfully relearn daily life skills and sustain these abilities over an extended period [80]. Additionally, earlier investigations explored the ability of dementia patients to recall familiar faces [81], name objects, and describe their uses [82]. One study, which also aimed to enhance memory for familiar faces, suggested that EL is an efficient method yielding substantial improvements in memory [41,42]. These findings align with the existing literature, demonstrating the effectiveness of errorless learning when compared to other approaches, such as error-prone learning, within patient populations [83,84,85]. Moreover, it is worth noting that the aforementioned studies did not employ technology-based intervention programs. Instead, they established a strong foundation for the development of more efficient tools for the cognitive treatment of dementia. Later studies emphasized the need for the use of computer-based technology in cognitive intervention along with the application of EL method and provided positive results from the combined application of both, suggesting that the EL approach is highly effective for enhancing and promoting memory abilities [43,86]. A study of Chinese-speaking dementia patients found that early AD subjects had an improvement in their overall cognitive function after attending the EL memory program, although the positive treatment effect had a limited duration [87]. It was further supported that older normal adults can benefit from an EL program, as part of a preventive strategy in order to minimize the risk of developing dementia. 

Finally, the results of various studies comparing the effectiveness of errorless and trial-and-error methods in acquiring knowledge, ranging from very general to very specific, were presented for both individuals with dementia and healthy participants [88]. It was concluded that the errorless learning (EL) approach appeared to be advantageous for retrieving specific knowledge, but less so for acquiring general knowledge. Furthermore, they noted that not all patients exhibited a consistent response to the learning conditions, suggesting limited applicability of this approach for patients with dementia and restricted efficiency for rehabilitating a broad spectrum of knowledge. These findings warrant further research.

### 2.4. Studies on Prospective Memory (PM) through Technological Applications

Prospective memory is the ability to remember to perform previously planned actions or certain tasks in the future [89]: for example, to remember and go to an already scheduled appointment, or to remember to call someone and wish for his/her birthday, or to remember to take their medication and so on—the so-called instrumental activities of daily living that ensure independent living. Prospective memory, in effect, comprises two components: the prospective component and the retrospective component. The first one has to do with remembering an action that must be carried out at a given time while the second one has to do with informational content, which must be retrieved, i.e., what we already know. This type of memory relies on other cognitive functions such as working memory, divided attention, executive functions, all of which show age-related deficits. Although prospective memory refers in effect to everyday memory and has a range of consequences if it fails, it remains an area of research that only recently has attracted more scientific interest [89]. So far, only few studies have been conducted and investigated more thoroughly the impact that this type of memory has on the demented patients’ daily living.

It is widely recognized that issues related to prospective memory are prevalent in aging [90], with these challenges being particularly evident in individuals with dementia [91]. Considering that aging represents the primary risk factor for developing dementia [92], there is a substantial need for the development of innovative and effective interventions to aid older adults in remembering their daily activities. These interventions encompass cognitive training techniques, such as intention implementation [93], as well as the utilization of uncomplicated technical devices to facilitate medication reminders [94].

A further distinction of the prospective memory has been made between event and time-based prospective memory [95]. According to this distinction event-based prospective memory involves remembering to perform a certain action when an external cue is given, such as remembering to phone someone when a picture of him/her is provided [55]. Time-based prospective memory involves remembering to perform a certain action at a specific time or after certain time has passed, for example taking medication ten minutes after lunch [55]. Furthermore, in [96], a distinction is made between pulse intentions, which require execution at a specific time, and step intentions, which have a more flexible timeframe for completion (e.g., “I need to call the bank at some point today”). These distinctions hold significance in clinical assessments.

To this end, the studies assessing prospective memory compare performance of older healthy adults to that of AD patients or traumatic brain injury (TBI) patients in a variety of tasks. Nevertheless, there is a scarcity of studies comparing the performance of patients suffering from other forms of dementia to that of healthy subjects, or investigating prospective memory in patients with dementia independently, except for the research presented in [77].

#### 2.4.1. Prospective Memory and Healthy Older Subjects

There are a limited number of studies so far that have addressed the issue of whether there is a link between prospective memory and daily living activities and quality of life in older adults, or the way that age related decline of prospective memory can affect the daily function of the elderly. In this line of work, it was found that the application of a computer game (the Virtual Week task) for the assessment of prospective memory, significantly aided performance of healthy older adults on related tasks [97]. Similarly, [98,99] observed a general enhancement in the prospective memory of the participants, indicating positive advantages of this training approach. In a systematic qualitative analysis, coupled with a quantitative meta-analysis on the impact of prospective memory (PM) training in older adults, it was indicated that there was a notable but modest immediate effectiveness of PM training in enhancing PM performance. However, no significant long-term effectiveness was discerned [100]. Similarly, reviews proposed that not only the specific training strategies employed for the assessment of prospective memory need to be further examined, but also certain parameters need to be considered, such as the participants’ digital literacy as well as their motor and sensory restrictions [101,102].

#### 2.4.2. Prospective Memory: Comparison of Healthy Subjects and Subjects with Dementia

In one of the first studies that compared healthy controls to mildly affected AD patients and patients with dementia in a range of prospective memory tasks [37], it was proposed that on prospective memory tasks the performance of individuals with mild AD was similar to that of patients with dementia. In a later study, event-based and time-based intentions were examined in participants with AD and in healthy controls [38]. The researchers discovered that individuals with Alzheimer’s disease (AD) exhibited poorer performance compared to healthy controls in recalling both time-based and event-based intentions. Consistent findings were reported in another study where patients with dementia demonstrated difficulties in their prospective memory, especially in the context of event-based tasks [39]. In a study involving 14 patients with memory impairments who were assessed in two types of prospective memory tasks (event-based PM and time-based PM), each under errorless learning (EL) and errorful encoding (EF) learning conditions, the results indicate an advantage of EL for the EBPM task but not for the TBPM task [77]. Problems with time-based intentions and event-based intentions are further supported in a more recent study [40].

Finally, it was found that the introduction of a memory encoding strategy could improve prospective memory in healthy older adults and in patients suffering from AD [103].

## 3. Materials and Methods

### 3.1. Participants

Thirty subjects overall participated in our study. In total, 15 of them with a chronological age of 63 to 85 years old were diagnosed with dementia, and the other 15 subjects were their matched controls for age, sex, and educational level. From the participants with dementia, 6 were men and 9 were women; 8 of them were diagnosed with AD, 4 with vascular dementia, 1 with frontotemporal dementia, 1 with dementia with Lewy bodies, and 1 with a non-specific type of dementia. Following [58], we applied similar exclusion criteria to our sample. These were: (1) brain tumor or encephalitis, (2) mental illnesses within two years before the start of the study, (3) severe depression, (4) Parkinson’ s disease, Huntington disease, (5) cases of medical diseases (e.g., thyroid disease) that seem to affect cognitive performance, (6) patients with alcohol or drug addiction, and (7) patients with motor impairments or any other physical disability that could hinder their participation in the computer-based tasks.

#### Participants Recruitment

The participants were recruited from different places in Greece, Athens, Achaia and Biotia for a period of 10 months. The patients either visited daily care centers or they were staying at a nursing home, permanently. The researchers informed the relatives of the patients and the staff of the daily centers and the nursing homes about the procedure and the purpose of the study, as well as the place and the time that the study would be carried out. After obtaining the subjects’ and their relatives’ consent, the researchers carried on with the administration initially of the screening tools in order to assess patients’ overall cognitive level, then with the questionnaire in order to pinpoint areas that needed intervention, and finally with the computer-based tasks that aimed to assess certain cognitive functions namely short-term memory and prospective memory.

### 3.2. Initial Assessment—Screening Tools

In order to assess the level of severity of their illness we initially administered to the participants with dementia the MoCA test which is standardized in the Greek population and is translated in the Greek language as well. This test was preferred over the MMSE since it assesses more cognitive areas and is more sensitive in the assessment of mild cognitive impairments [104].

In addition to MoCA test, a questionnaire was also developed and administered to the participants with dementia in order to investigate a number of aspects. These included information about their gender, marital status, education, living conditions, medication and the frequency that they take their medicines, the frequency they visit their doctors, the frequency of their relatives’ visiting, management of their finances, nutrition habits, complaints about their memory gaps, and acquaintance with any technological devices. Overall, 23 questions were developed and given to the patients. The results from the questionnaire showed that the patients were not well acquainted with technological devices. However, they also indicated four main problematic areas for the patients: object/place association, familiar face recognition, nutrition, and medication intake.

The amount of time that was needed for the participants to complete the questionnaire was 15–20 min. In cases where a caregiver was responsible for the patient, they also had to complete the questionnaire to ensure a match between the patient’s responses and those of their caregiver. The instructions on this questionnaire were given to the participants orally and their answers were transcribed by the researchers.

Based on the overall findings from the questionnaire a further study was conducted targeting the four specific areas that were found to be problematic for the patients. This was succeeded through the design of tasks that were based on a technological device (tablet, computer).

### 3.3. Experimental Design

The findings from the initial assessment indicated that the patients were not highly acquainted with any technological devices. At the same time, it was revealed that there were four areas of difficulty that warranted further investigation. These areas included: the ability to remember to locate various things within certain places, the ability of face recognition, the ability to remember to feed themselves, and the ability to remember to take their prescribed medications when needed. These four problematic areas illustrate, in effect, problems with short-term memory (allocation of things within certain places and face recognition) and with prospective memory (intake of their food and of their medication), respectively. Thus, two tasks were presented to the patients—one for short-term memory and one for the prospective memory. Each task had two activities with their respective stimuli-sets which were presented using the errorless learning method in order to enhance patients’ performance. Prospective memory was assessed through time-based tasks in which the participants were prompted to remember, based on the time presented to them, the appropriate action (i.e., taking medication or eating a meal) while pictures/phrases with the respective stimuli were depicted.

During the intervention period patients and healthy controls participated in six sessions each lasting approximately 30 min. There was a step-by-step procedure adopted based on the ABA principles of behavior. In this respect, there was a practical guide given to the participants from the start of each task, having a step-by-step analysis of what the participants had to do until they completed each task. The researcher recorded whether the participant needed guidance during each session. Full guidance (i.e., both verbal and physical) was marked with 1 point, partial guidance (i.e., only verbal guidance) with 2 points, and no guidance at all with 3 points.

The stimuli used in the tasks were presented in both auditory and visual format to minimize further demands on short-term memory, but also to minimize any possible sensory limitations that could affect participants’ overall performance while prompting at the same time the participants to provide the correct answer. To our knowledge, the manner of stimuli presentation (using both modalities) has not been employed in any other study so far in Greek language.

In each session, the participants (both patients and their healthy counterparts) were encouraged to avoid guessing the correct answer, since this was provided to them in the case of an error. Moreover, to ensure that there would be no training or familiarization effects, the stimuli of each task were presented to the participants in a random order. In addition, following the basic principles of errorless learning, in each task the stimuli were divided into smaller steps of graded difficulty.

Overall, the activities that were selected and included were tailored to the needs of the specific participants, namely allocation of things in specific places (i.e., object/place association), face recognition, medication times, and mealtimes. Despite the participants’ limited familiarity with technological devices, this study presented the stimuli in a user-friendly manner to encourage greater utilization by the patients.

The application was created using PowerPoint 2016 and utilizes hyperlink navigation along with Visual Basic for Applications (VBA) scripting. Hyperlink slide navigation enables the generation of interactive links that direct users to specific slides within the presentation. The slides within the PowerPoint presentation were designed as questions, each accompanied by multiple-choice answers. For object/place association questions, there are five levels in total. The 1st level presents a single answer option, the 2nd level offers two possible answers, the 3rd level provides three multiple-choice answers, and so on, up to level 5. In the event of an incorrect choice, the hyperlink redirects to the correct answer in line with the errorless learning strategy. Conversely, if the user selects the correct answer, the hyperlink directs them to a new question.

For the task of prospective memory, a similar procedure was followed with varying time targets for training. Given a particular time, slides with multiple choice answers were used with a maximum of 3 choices. The choices were time slots of morning, midday, and nighttime. Once a correct choice of time slot was made, the hyperlink led to a multiple-choice slide with medication choices (pillbox choice) or meal choices (breakfast, lunch, dinner), depending on the activity. In the event of an incorrect answer for the time slot, the hyperlink led to the correct one, in accordance with the errorless learning strategy. Similarly, in the case of a correct time slot but incorrect medication/meal choice, the hyperlink, according to errorless learning, led back to the correct medication/meal choice. In addition, for this particular activity, there was an option provided for displaying the actual computer time on a slide and then navigating to the appropriate slide based on the time of day using VBA scripting. This was incorporated considering the potential for a real-time application.

#### 3.3.1. Task 1 Short-Term Memory—Stimuli and Procedure

Before each session, two examples were provided for the object/place association activity in order to acquaint the participants with the procedure. The first part of each session was dedicated to the short-term memory task which lasted approximately within 20 min.

**Figure 1:**
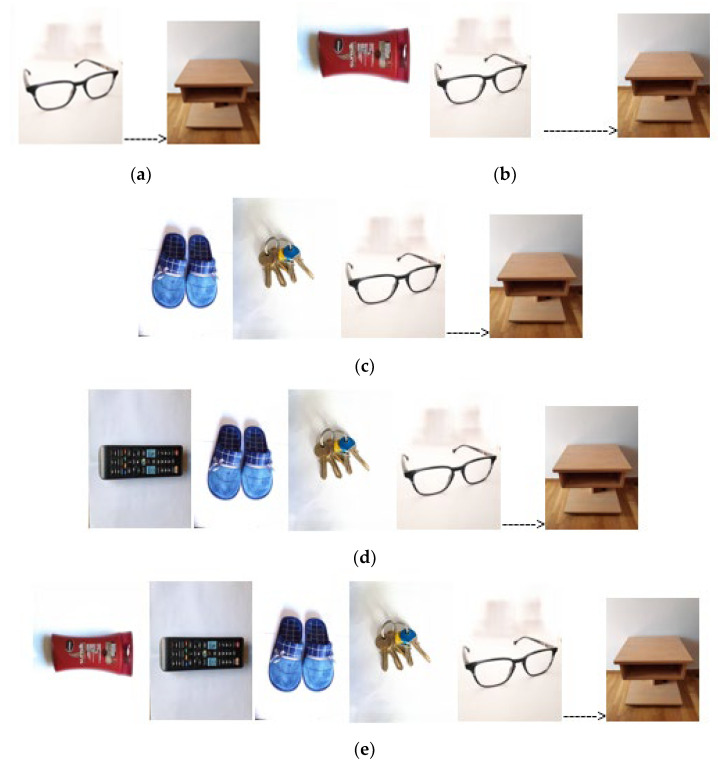
Example of five levels of object/place association: (**a**) level 1—one object (target: object glasses, place nightstand); (**b**) level 2—choose from 2 objects; (**c**) level 3—choose from 3 objects; (**d**) level 4—choose from 4 objects; (**e**) level 5—choose from 5 objects. On the left side of the arrow are the objects, while on the right side is the target place.

With respect to object/place association, the activity was presented in graded difficulty starting from one object associated with one targeted place (Figure 1a) up to five objects and one place (Figure 1e).

It should be noted that prior to the initiation of the experimental procedure, all stimuli of the object/place association activity were presented to the 15 healthy controls to assess whether the objects and places were easily recognized/identified (naming). Stimuli that were not identified by all participants were excluded.

In the face/name association activity, participants were presented with pictures of people from their environment and name association was performed based on the most prominent face feature, e.g., Nikos has a mustache. Once more, this activity presented a graded difficulty where the participants had to select from one face and one name to five faces and one name.

#### 3.3.2. Task 2 Prospective Memory—Stimuli and Procedure

In this task, the researcher presented participants with a specific time and they were required to determine which of three time slots it corresponded to: morning, midday, or nighttime. Each time slot was associated with either a colored medication pillbox (medication activity) or a meal name, such as breakfast, lunch, or dinner (meal activity). The actual time slot, such as 9–11 or 8–10, was customized for each participant.

Consequently, the sequence of actions involved in these activities included identifying the presented time, selecting the appropriate time slot, and ultimately associating it with either a medication pillbox or a meal name. If a participant made an incorrect selection at any step, they were guided back to the last correct step, provided with correct answer feedback as part of an errorless learning approach, and then allowed to proceed to the next step. For instance, if a participant was given the time 9:30 am, they first had to determine it was morning and then associate it with the corresponding meal, such as breakfast. The total time allocated for completing this task was approximately 10 min.

### 3.4. Statistical Methods

The Wilcoxon signed-rank test was employed to compare the maximum performance of dementia patients with that of healthy controls. Additionally, the Mann–Whitney U test was utilized to assess differences between the sexes within both groups. Furthermore, Spearman correlation tests were conducted to examine the relationships between MoCA test results and the performance on app tasks, as well as between age and the performance on app tasks; these were carried out for both patients and healthy controls.

## 4. Results

The results from the two samples with respect to MoCA and their performance in the computer-based tasks are illustrated in Table 1.

There was a statistical difference (*p* < 0.001) between healthy controls and patients with dementia with respect to their performance in the computer-based tasks across the four areas that were initially suggested to pose difficulty to the patients. Indeed, it seems that the overall performance of the patients is worse than the performance on the healthy controls in the relevant tasks.

The results of the Mann–Whitney U test showed no statistical difference in the performance between men and women of both our patient sample and healthy sample, in all tasks.

With respect to patients’ performance in the MoCA test, it was observed that the worse their score was, the worse was their performance in the computer-based tasks. In addition, age was also an indicative factor that affected performance, i.e., the younger the patient, the better their performance. These results are shown in Table 2 where significant correlations are indicated by asterisks.

The application of errorless learning seems to have had a positive effect on the performance of the participants, especially of the healthy controls and to a lesser degree to that of the patients. It was observed that there was no significant improvement from one session to another but there was an overall improvement in patients’ performance that was clearly illustrated in the final sessions as compared to their performance in the initial sessions. It is also important to stress that there were no training or familiarity effects with the way that the material was presented, since this was given in each session in a random order.

For healthy controls, however, the results are more encouraging since it was observed that there was an improvement throughout each session and through the completion of all sessions over the time. Remarkably, 54% of healthy controls were able to perform each step of the given task with some or no guidance at all, from the second session already. This outcome is in line with several studies with older people [105,106,107,108], suggesting that computerized cognitive intervention and training can lead to significant improvement of various cognitive functions such as short-term memory, working memory, visual memory, etc.).

## 5. Discussion

In light of the extensive body of research in this field, our objective was to create a user-friendly computer-based application aiming to enhance short-term memory and prospective memory abilities in both healthy older individuals and those with dementia.

The results from our study showed that patients faced more difficulty than the older healthy adults across a number of tasks that concerned allocation of things in specific places, face recognition, medication intake, and food intake. Failures in both types of memory have been noted in earlier studies, compromising independence in daily living [89,92,94]. The difficulties in the patients’ performance observed in the current study were highly associated with their overall cognitive decline as this was indicated by the screening tests. That is, the lower the score, the greater the memory difficulty; a finding which is not surprising as there is a more rapid decline as a result of each patients’ neurological disease. However, memory difficulties were less evident in the group of the healthy participants. A superior performance of healthy controls as compared to patients suffering from dementia is a finding demonstrated in various studies [38,39,40]. This was further supported in the current study as well, reflecting presumably a neural decline that is evident in dementia rather than a failure to respond to the demands of the tasks employed. The involvement and decline of particular brain networks in dementia has also been well-identified [71,72,73,74] and shown to affect patients’ performance in the relative memory tasks.

The use of the errorless learning method as an intervention for enhancing and retaining short-term memory and prospective memory abilities in our dementia subjects has shown effectiveness only during each individual session. However, at the end of the training program we noted an overall improvement to our patients’ performance as compared to their performance before the intervention. These findings are encouraging with respect to the positive effects that a computerized program can have, as this is captured by the patients’ improvement. In a study where participants had mixed diagnoses, as in our study, and errorless learning method was used, it was found that this was effective only in some of their patients [88]. In other studies, patients had an improvement in their memory skills [43,87]. Nevertheless, since our study is one of the initial attempts in the Greek population that aims to address the effectiveness of this method, no solid conclusions can be drawn. More research is needed to shed more light with respect to the intervention means and techniques that must be developed to produce clearer and long-standing outcomes.

We think that another point that is of some value, but surely needs further investigation, is the way that the stimuli was presented to our participants. Both visual and auditory modalities were used in order to minimize memory difficulties and enhance the encoding procedure without having the patients resort to any recalling strategy, as this was compromised. Even in that case, the improvement of our patients was slow and apparent only at the end of the training program. The benefits of these methods need more thorough research before drawing conclusions and generalizing their application.

We turn now to one more issue, namely the capability and willingness of older individuals, either healthy or affected by some kind of a neurological disease, to use new technological devices. In a longitudinal study, it was stressed that older healthy individuals would be keen on using any electronic device that would assist them with their memory problems such as prospective memory problems as long as this would not be very expensive and as long as it would be easy to use on a daily basis [109,110,111]. A recent study reported that the participating older adults that followed a computerized cognitive training program mentioned that their exposure to this computerized program was a positive experience regardless of their computer literacy skills [112]. The importance of using attainable, yet challenging, material, as well as the need to reduce frustration to the participants ‘with strategy use and assistance via supervised sessions’, was noted. It is suggested that assistive technology can make a difference in the lives of people with dementia and to their caregivers as well [113]. This is especially true when technologies can be part of a home package assisting people to maintain their independence and improve the quality of their life. Bjørneby et al. [114] describe the main principles of user requirements, suggesting that technologies should support someone’s choices, affect positively his/her life, support skills maintained and not emphasize lost skills, focus on the abilities and not on the disabilities of the user, and provide a sense of independence to the person. In an era in which great technological advances have been achieved, it is of great importance to provide people with devices that can help them attain their daily functioning and autonomy, and if possible, personalize their use in order to meet each person’s needs. Another study [115] proposed that when technology is applied to patients suffering from dementia, a number of parameters must be taken into consideration: usage of large, well-contrasting icons; implementation of gesture control; personalization of the material used; keeping cognitive requirements to a minimum; the ability to adjust difficulty levels to each patients’ current cognitive and physical condition; in dual-tasking applications, one cognitive skill should be combined with one motor skill; and finally implementation should be as part of a supervised group activity. A review paper explored the effects of smartphone and tablet computer use on cognition and memory in older healthy adults and in adults that face cognitive impairments [102]; barriers to smartphone and tablet use were reported. It concluded that these devices are currently underutilized as a means to support a number of cognitive processes and that their use can indeed enhance some cognitive functions, but not to the same extent with memory. It also determined that ‘there is a lack of gerontological smart device based research’ and that there is a need for further research into older adults’ spontaneous smartphone and tablet use.

In accordance with these observations, we suggest that the application of technological devices in the area of rehabilitation can assist patients in improving the quality of their lives on a daily basis as long as this respects their physical and cognitive limitations. To circumvent potential difficulties derived from patients’ organic problems, we presented our testing material in a simple and clear way, targeting both the visual and auditory modality and taking into consideration the needs of our patients. It is obvious, however, that further research needs to be carried out in order to develop more appropriate technological techniques and subsequent testing material tailored to each patients’ needs. At the same time, with the advances in the area of neuroimaging, more information can be gained about the neural substrates involved in dementia prompting; thus, the development of more solid theories about the structure of memory and more appropriate rehabilitation strategies are needed.

### Limitations

A limitation of the current study is the small sample size, rendering the generalization of the findings limited. As this study serves as a pilot investigation, the results provide preliminary indications that warrant further evaluation of the effectiveness of the current app in a larger clinical sample.

Another limitation is the absence of a follow-up assessment to determine whether the observed cognitive improvements among both patients and healthy adults are sustained over time. Additionally, there is no assessment of any potential transfer effects of these cognitive enhancements to daily life activities.

On the other hand, the merit of this study is to lay the foundation for further research regarding the implementation of nonpharmacological approaches through technological devices for the improvement of cognitive functions that decline either due to normal aging or cognitive impairment. The steady improvement of cognitive functions, particularly among healthy older adults, raises hope in the efficacy of computerized cognitive training when delivered in a user-friendly manner. Considering the scarcity of Greek-language studies addressing computerized cognitive treatment approaches, the outcomes of this study provide encouragement for conducting further research in the field of cognitive impairment treatment.

## 6. Conclusions

The current study aimed to develop a pilot app that could respond to the patients’ needs. Of course, this was neither an extensive nor a longitudinal study that could assess the effects of technology not only in a short but also in a long-term basis and adjust further its methods in order to achieve better outcomes in respect to our patients’ cognitive functioning. However, it constitutes an initial attempt to address the issues outlined in the current literature of dementia and lays the groundwork for further research of the healthy and neurologically impaired aging Greek population. We do hope that further research will shed more light on our understanding of the neurological underpinnings of the cognitive functions that decline when the brain is affected by some neurological condition. This, in turn, will enable us to construct the appropriate tasks with the appropriate methodology and develop the necessary equipment to improve patients’ everyday living.

## 7. Recommendations

Further research is required to conduct a long-term follow-up in order to enhance our understanding of whether the implementation of CCT programs can positively impact targeted cognitive domains and simultaneously investigate the durability of these gains over an extended period post-training. Moreover, additional studies could prioritize the investigation of other facets of fluid intelligence, such as executive functions, which appear to play a pivotal role in global cognitive decline [50]. It is also imperative to maintain continuous evaluation of both pharmacological and non-pharmacological treatment interventions to develop more sophisticated strategies for addressing the constellation of symptoms evident in dementia, including cognitive, behavioral, and psychosocial symptoms. However, achieving this goal necessitates larger-scale samples and a greater number of trials. Furthermore, it is crucial to address inconsistencies among the various methodologies employed in different studies. Additionally, the utilization of more suitable outcome measures capable of assessing the potential generalizability and persistence of any effects should be considered [116].

## Figures and Tables

**Table 1:** Patients’ and matched healthy controls’ performance: MoCA and computer-based tasks (*N* = 15).

	Patients		Healthy Controls	
Variable	Range	Mean (SD)	Range	Mean (SD)
Age	63–85	76.53 (6.62)	67–83	75.29 (5.40)
MoCA *	5–20	11.87 (4.39)	25–30	27.36 (1.50)
Object/place relation *	12–28	17.07 (5.20)	23–28	25.50 (1.61)
Medication *	13–24	17.00 (3.91)	23–29	26.71 (1.86)
Nutrition *	12–28	17.53 (4.41)	23–29	26.79 (1.76)
Faces *	11–28	17.47 (5.18)	24–30	27.21 (1.93)

* Significant difference at the 0.001 level in the Wilcoxon signed-ranks test.

**Table 2:** Spearman correlation results between MoCA and computer-based tasks (both groups).

	Object/Place Relation	Meditation	Nutrition	Faces
MoCA (patients with dementia)	0.687 **	0.522 *	0.695 **	0.719 **
MoCA (healthy controls)	0.911 ***	0.966 ***	0.905 ***	0.779 ***
Age (patients with dementia)	−0.700 **	−0.476	−0.414	−0.293
Age (healthy controls)	−0.819 ***	−0.877 ***	−0.877 ***	−0.81 ***

* Correlation is significant at the 0.05 level. ** Correlation is significant at the 0.01 level. *** Correlation is significant at the 0.001 level.

## Data Availability

The data are not publicly available due to privacy and ethical restrictions.
